# Early Experience with the Subcutaneous ICD

**DOI:** 10.1007/s11886-014-0516-1

**Published:** 2014-07-04

**Authors:** Pier D. Lambiase, Neil T. Srinivasan

**Affiliations:** The Heart Hospital, University College Hospital & Institute of Cardiovascular Sciences, UCL, 16-18 Westmoreland Street, London, W1G 8PH UK

**Keywords:** Internal cardiac defibrillator, Sudden cardiac death, Ventricular arrhythmia, Defibrillation, Subcutaneous ICD

## Abstract

The Subcutaneous Internal Cardiac Defibrillator (S-ICD) represents a major advance in the care of patients who have an indication for an internal cardiac defibrillator without pacing indications. Its main advantage is that it can deliver a shock to cardiovert ventricular arrhythmias utilising a tunnelled subcutaneous lead, negating the risks associated with conventional transvenous systems. Initial studies have shown comparable efficacy in cardioversion of induced and spontaneous ventricular tachycardia (VT) and ventricular fibrillation (VF) when compared to conventional transvenous systems. In addition, inappropriate shocks occurred in a similar percentage of patients to conventional ICD studies. Complication rates are low and relate largely to localised wound infections, treated successfully with antibiotics. The long term efficacy of the device is yet to be ascertained, however, a randomised trial & prospective registries are currently in progress to enable direct comparison with transvenous ICDs. This article summarises the early clinical experience and trials in the implantation of the S-ICD.

## Introduction

The Subcutaneous Internal Cardiac Defibrillator (S-ICD) represents a significant technological advance in the prevention of sudden cardiac death avoiding the need for an intravascular lead in ICD recipients without pacing indications [1–3].This is a particular advantage in young patients who can then avoid chronic lead complications of fracture & insulation breaks, patients with vascular access issues (paediatric, congenital heart disease, venous occlusion) or high risk of bacteraemias, e.g. renal dialysis & chronic indwelling catheters. Following initial approval in Europe in 2009 and the FDA IDE study in 2012 [4••], this technology is being employed clinically worldwide. Initial small single centre experience is being built upon with ongoing data collection from IDE study patients and the EFFORTLESS (Evaluation oF FactORs ImpacTing CLinical Outcome and Cost EffectiveneSS) S-ICD Registry in Europe [4••, 5••, 6••]. The latter is the first ICD device study to collect detailed implant and follow-up data in 1000 patients up to 5 years post procedure providing real world experience outside the usual remit of randomised controlled trials. In this review, the current evidence relating to S-ICD system performance & outcomes plus issues of optimal patient selection will be discussed.

## Overview

**Fig. 1:**
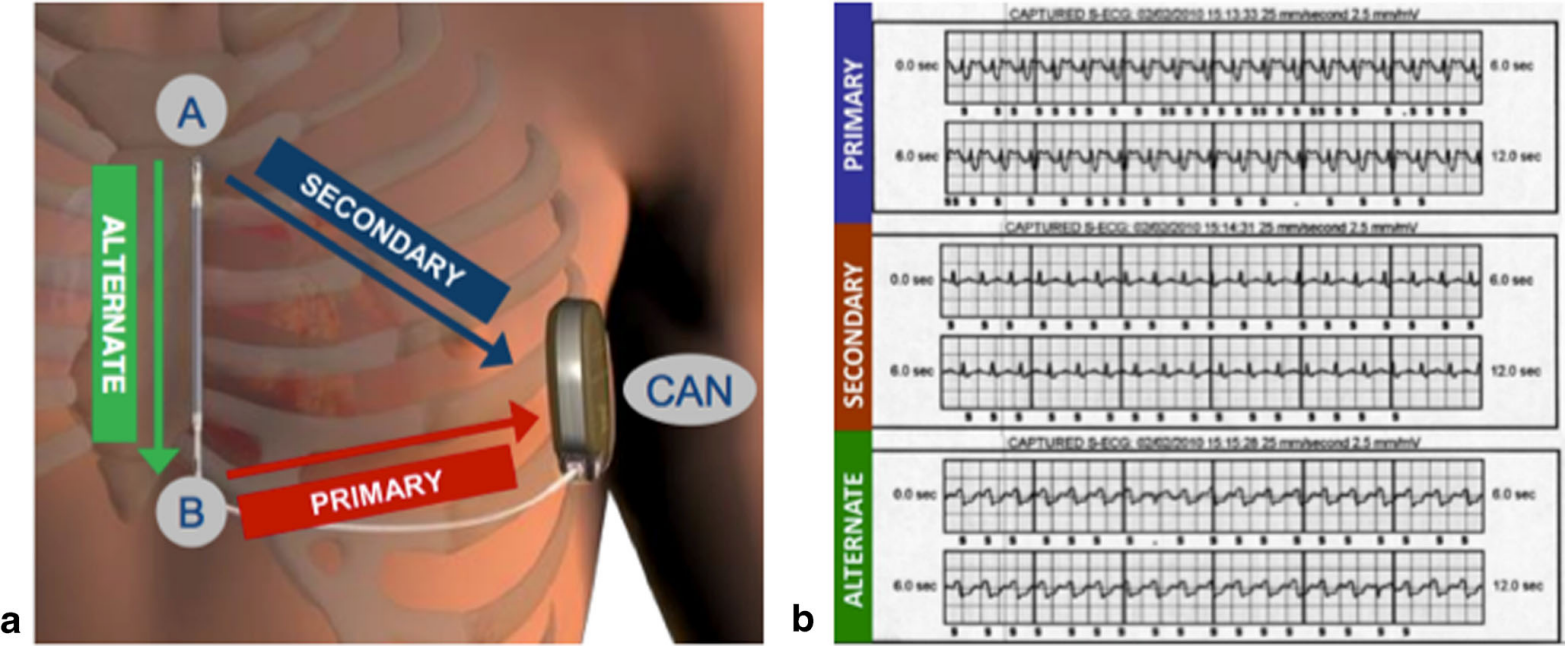
**a**) The three shocking vectors that can be used by the device. **b**) Inappropriate T-wave oversensing in the primary and alternate vector during exercise, with correction by use of the secondary vector

The details of the S-ICD system and implantation technique have been described elsewhere [2, 7•]. In summary, the device is placed in the left lateral position via a lateral submammary incision and the lead tunnelled to be positioned parasternally sensing the surface ECG electrogram in three vectors (Fig. 1). Advances in sensing algorithm technology and programming have facilitated optimisation of surface ECG sensing to minimise inappropriate shocks for sinus tachycardia and rapidly conducted atrial fibrillation (AF). These advances have mainly arisen through software developments in signal processing and optimisation of programming [8]. The device has two programmable zones of tachycardia detection, a supraventricular tachycardia (SVT) discrimination zone and a ventricular fibrillation (VF) zone. The latter is purely dictated by ventricular rate whilst the SVT discrimination zone utilises a number of parameters including electrogram morphology and stability to differentiate between ventricular tachycardia (VT)/VF and SVT. Therapy is then withheld if SVT discrimination criteria are met below the VF therapy heart rate threshold. These algorithms can be ineffective if the patient develops bundle branch block during SVT, although this can be overcome by employing an electrogram template recording aberrant beat morphology if it has been recognised as an issue during screening. However, major challenges remain with T wave oversensing and rapidly conducted AF into the VF zone when only a rate criterion is applied. T wave oversensing can be minimised by optimising the sensing vector prior to implant and performing an exercise test examining this vector(s) to determine whether this will be an issue risking inappropriate shocks [8, 9] (Fig. 1). A recent paper from the Dutch group evaluated the factors most likely to cause a failure in ECG screening for the S-ICD [10•]. They examined patients who did not have pacing indications and accepted only those patients that had at least one suitable sensing vector for distinguishing QRS complexes and T waves. On this basis, they found 7.4 % of cases (all male) did not have suitable sensing vectors in the lying and standing position, the main factors responsible for ECG screening failure being hypertrophic cardiomyopathy, increased BMI, a broad QRS, and an R:T ratio of <3 in the ECG lead with the largest T wave. This screening failure rate is relatively low, but it may be possible to address this with more tailored algorithms in the future or by using more bespoke specific separate sensing electrode placements to minimise this problem in certain cases. These electrodes could be separate from the shocking coils, although it is already being recognised that in more centrally positioned hearts one can place the ICD electrode in the right parasternal position as opposed to the left, achieving optimal sensing and effective cardioversion of induced VF if necessary.

## Efficacy in the Cardioversion of VF

The critical concerns of the S-ICD from both a clinical and regulatory perspective have been both the prompt detection and conversion of VF. This has been carefully addressed both in initial phase 1 studies of the system and formally in the IDE trial as well as EFFORTLESS Registry [2, 4••, 5••]. Together the latter represent data on just over 800 patients who have been implanted with the system with smaller series reported from Netherlands, UK & Germany (Table 1) [11•, 12, 13]. Since the larger IDE & EFFORTLESS cohorts represent more recent experience with adequate population numbers to enable meaningful conclusions on system performance to be drawn, the discussion will focus primarily on these two large studies and refer to the smaller series where appropriate.

**Table 1:** Main published S-ICD cohorts

Study population	n	Case mix	Mean age	Appropriate shock	Inappropriate shock	Complications	Mean Cumulative	
		1° prevention	(y)	rate	rate	overall	FUp/pt	FUp
				(%Pts)	(%Pts)	(%Pts)		
Bardy et al. 1st European Trial	55	78 %	56 ± 13	5.5 %			10 months	46 pt-years
IDE	314	79.4 %	51.9 ± 15.5	6.7 %	13.1 %	7.9 % at 180d	330d	289 pt-years
EFFORTLESS	472	63 %	49 + 18	7 %	7 %	6 % at 360d	558 d	721 pt-years
Dutch Cohort Study	118	38 %	50 + 14	7 %	13 %	11.9 %	18 m	177 pt-years
German Initial Cohort	40	42.5 %	42 ± 15	10 %	5 %	Nil	229 d	25 pt-years
UK Survey	111		33 (median)	12 %	15 %	1 arrhythmic death	12.7 ± 7.1 months	117 pt-years

## Induced VF

The IDE trial was a prospective, non-randomised, multicentre trial which studied adults with ICD standard indications who neither required pacing nor documented pace-terminable ventricular tachycardia [4••]. The primary effectiveness end point was the induced VF conversion rate compared with a pre-specified performance goal of 88 %. These successful shocks had to be two consecutive VF conversions at 65J in either shock vector, within a maximum of four VF conversion attempts with the use of the same polarity to qualify as a successful DFT. An additional very stringent analysis was performed in which patients in whom VF inductions could not be completed for technical or clinical reasons were classified as failures, e.g. inability to induce VF or the clinical status of the patient deemed by the implanting physician made testing unsafe. The primary effectiveness cohort consisted of 304 patients who completed the full testing protocol, providing evaluable conversion tests according to the protocol definitions. The conversion rate in these evaluable conversion tests demonstrated 100 % acute conversion with a 95 % lower confidence limit of 98.8 %, which exceeded the pre-specified objective performance goal of ≥88 %. In a total of 265 patients (82.8 %), acute conversion of VF was successful on consecutive shocks one and two in the first polarity tested after the final position was achieved. Sixteen patients were deemed non-evaluable and one patient who did not undergo any testing because of persistent left ventricular thrombus. Including these 17 excluded patients as VF conversion failures reduced the acute VF success conversion rate to 94.7 % with a 95 % lower confidence limit of 91.7 %, still exceeding the pre-specified performance goal of 88 %. This data is very similar to that reported in the initial analysis of EFFORTLESS which reported on induction testing in 393 patients with complete data [6••, 14]. Due to the variability in acute defibrillation testing protocols in the latter at each clinical site, successful conversion efficacy at implant was defined for the Registry as at least one successful conversion of an induced ventricular arrhythmia at ≤80J. In eight cases information was incomplete, while in nine patients VT/VF was not inducible. Of the 393 patients with complete data, in all but one patient VT/VF was successfully converted (99.7 %). Seven of these patients had an initial conversion failure that required one or more procedures to reposition the system to become successful. A shock energy of ≤65J was successful in 95 % of patients. Therefore, the acute conversion rate was similar and times to therapy were 15.1 ± 3.8 s (range = 7.0-37.0 s) for 65J shocks versus 14.6 ± 2.9 seconds, (range 9.6- 29.7 s) in IDE.

### Spontaneous VT/VF Episodes

The IDE study reported 119 spontaneous VT/VF episodes in 21 patients (38 discrete VT/VF episodes and 81 occurring during VT/VF storms). The 38 discrete VT/VF episodes consisted of 22 episodes of monomorphic VT (13 patients) and 16 episodes of polymorphic VT or VF (11 patients) [4••]. A total of 43 appropriate shocks were delivered in these 38 discrete VT/VF episodes, all of which terminated the arrhythmia. The S-ICD System converted 35 of 38 episodes (92.1 %) on the first shock and 37 of 38 (97.4 %) with one or more shocks. This compares to the EFFORTLESS data where 169 episodes received therapy in 59 patients. Of these, 93 episodes (55 %) in 33 patients were classified as VT/VF; 51 were discrete episodes (n = 29 patients) and 40 were episodes recorded during VT/VF “storms” (defined as ≥3 treated VF/VT episodes within 24 hrs) with two additional VT/VF episodes which spontaneously converted prior to first shock. Of the 51 discrete episodes receiving therapy, 45 converted to sinus rhythm either immediately or within a few seconds after the first shock (type 2 break, n = 3) giving a first shock conversion efficacy of 88 %. In the remaining six episodes, more than one shock was required to achieve cardioversion to sinus rhythm. The overall shock conversion efficacy per protocol definition of successful conversion within one device-defined episode and five shocks was 96 % (49/51 episodes). The mean time to therapy was 17.5 (±4.4) seconds with a range of 6.0 to 29.4 seconds reflecting a slightly longer charge time for the higher energy shock delivery in the ambulatory setting.

Six VT/VF storm events in four patients resulted in the 40 episodes in the EFFORTLESS cohort [6••]. One renal dialysis patient had multiple VT/VF storm events over a period of 17 months post implant and subsequently died due to pump failure. In one case of a patient with Loeffler’s syndrome, the VF storm was preceded by a 10 minute period of bradycardia (lowest heart rate of 28/min in the 60 seconds pre-arrest). The VF that subsequently developed was not successfully defibrillated, and the patient died. This unusual patient had obliteration of the RV and LV apices by a mass and was not deemed suitable for a standard ICD system. At implant VF had been sensed appropriately and cardioverted at 65J.

Other studies have reported equivalent data (Table 1 [11•, 12, 13]), but with no other failures to cardiovert VF. An age and sex matched study (n = 69) comparing defibrillation efficacy in S-ICD and transvenous ICD (TV-ICD) patients demonstrated a first shock efficacy of induced VF of 89.5 % using a 15 J safety margin compared to TV-ICD of 90.8 % (p = 0.8) increasing to 95.5 % with a second shock at reversed polarity [15]. In the UK survey study, there was one arrhythmic death which was thought to be due to a bradycardia. No other suspicious arrhythmic deaths have been described. Therefore, in the total of 1110 patients implanted to date it appears there have been two arrhythmic deaths with one due to failed conversion of VF in a patient whom the pathological process of eosinophilic infiltration could have caused an elevation in DFT. Concerns have been raised regarding the more prolonged detection and charge times seen in a proportion of S-ICD patients versus standard ICD systems. On average standard ICD times to detection and shock therapy are approximately 5 seconds shorter [16]. However, as discussed later these delayed detection and charge times may actually help reduce unnecessary shocks due to self-termination of VT/VF and even be a factor in reducing mortality in ICD recipients.

### Inappropriate Shocks

A well recognised issue in ICD function has been the problem of inappropriate shocks which range in incidence from 12-17 % in randomised controlled trials [17••, 18••, 19, 20]. There have been concerns that this will present a major hurdle to S-ICD uptake since the device relies on surface ECG electrograms as opposed to intracardiac signals for arrhythmia detection, making it more susceptible to error from T wave oversensing and electromagnetic interference (EMI) [9, 21, 22].

In the IDE trial, the incidence of inappropriate therapy was 13.1 % (41 patients) over the 11-month mean follow-up. Supraventricular tachycardia in the high-rate zone (no discriminators), in which rate alone determines whether a shock is delivered, was the cause in 16 patients (5.1 %) [4••]. Oversensing causing inappropriate shocks occurred in 25 patients (8.0 %); 22 patients experienced oversensing of T waves or, more rarely, broad QRS complexes, and in three patients as a result of external noise due to EMI from electrical equipment. Interestingly, as experience with dual zone programming increased in the latter 2/3 of patients treated in the trial, both T wave oversensing and SVT triggered inappropriate shocks showed relative reductions in incidence of 56 % and 70 % respectively. No patients suffered shocks due to discrimination errors in the SVT zone.

In EFFORTLESS, experience has been similar with 73 inappropriate shocks recorded in 32 patients over an average follow-up of 18 months (360 day inappropriate shock rate of 7 %). The majority of inappropriate shocks were due to oversensing (85 %) most frequently of cardiac signals (94 % of over-sensed episodes). In four patients, inappropriate shocks were due to noise or EMI while six patients had inappropriate therapy due to supraventricular arrhythmia (SVT) rates that crossed into the shock-only zone. There was one episode of discriminator error, in which morphology was impacted by a clipped signal. Therefore, both studies indicate that programming SVT zone (rate plus discriminators) significantly impacts on reducing inappropriate shocks from the device.

**Fig. 2:**
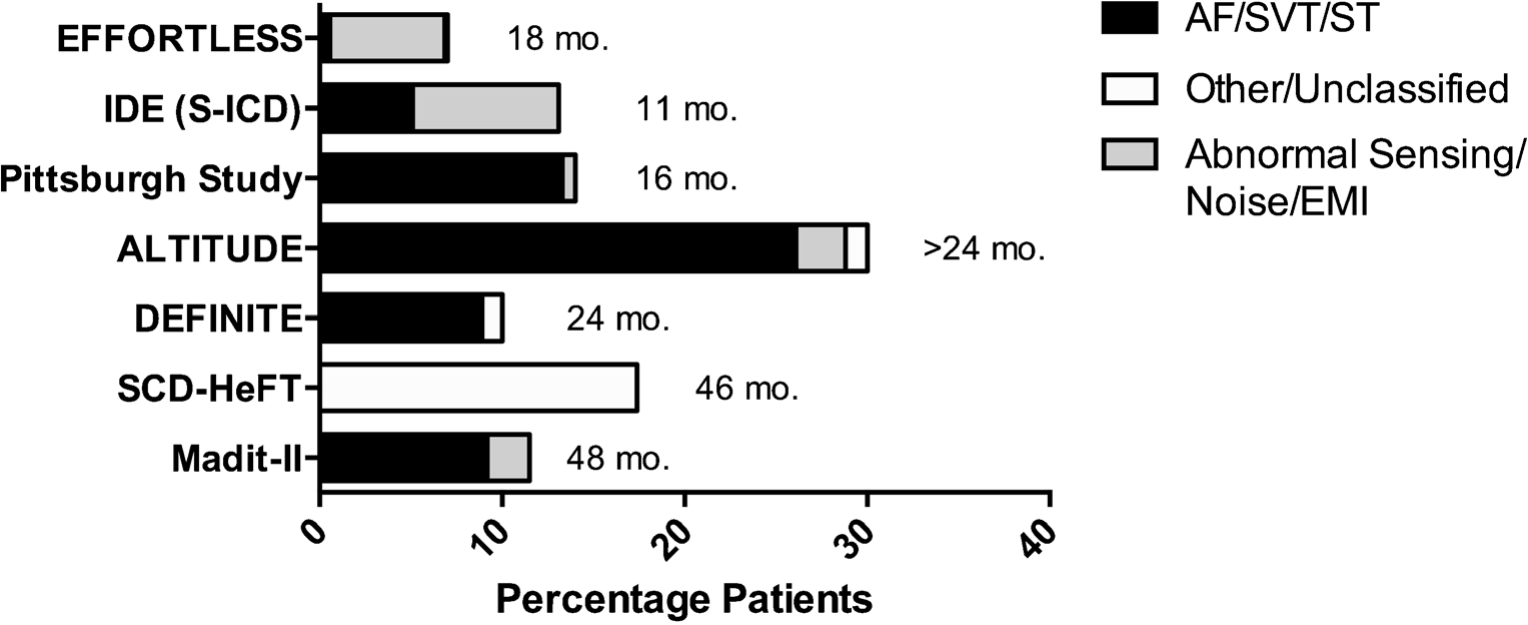
Inappropriate shocks, as a percentage of patients with ICD implanted in trials. AF-atrial fibrillation, SVT-supraventricular tachycardial, ST-sinus tachycardia. mo. is months of follow up. MADIT II is Multicenter Automatic Defibrillator Implantation Trial [19], IDE (S/C ICD) is Subcutaneous ICD IDE study [4••], SCD-HeFT is Sudden Cardiac Death in Heart Failure Trial [31•], DEFINITE is Defibrillators in Non- Ischemic Cardiomyopathy Treatment Evaluation [20], ALTITUDE is ALTITUDE Survival Study [30], Pittsburgh Study [33], EFFORTLESS [6••]

Figure 2 shows the inappropriate shock rate of the IDE and EFFORTLESS trials in comparison with transvenous ICD trials. It can be seen that the percentage of patients experiencing inappropriate shocks in the IDE trial is not dissimilar to experiences with conventional ICD implantation. Abnormal sensing was the major cause of inappropriate shocks in the IDE and EFFORTLESS studies when compared to other trials, however as experience has grown, the change to dual zone programming and optimising of screening to minimise T wave oversensing, will likely see a reduction in this number [9].

The device has been widely used beyond the standard ischaemic and dilated cardiomyopathy populations including patients with ion channel disorders (long QT syndrome, Brugada syndrome, catecholaminergic polymorphic VT (CPVT)), hypertrophic cardiomyopathy and congenital heart disease [23–26]. Indeed in the EFFORTLESS registry a significant proportion of cases had ion channel disorders and idiopathic VF. These disease sub-populations represent a significant challenge in terms of risks of T wave oversensing and inappropriate shocks due to increased heart rates as there are more frequent dynamic changes both in T wave and QRS morphology in these patients [27]. Furthermore, being younger than standard ICD recipients, they often achieve higher exercise heart rates which are at risk of putting them into a VF therapy zone if this is programmed too low, e.g. 200 beats/min. A number of these conditions especially CPVT cause increased ventricular ectopy and bigeminy which can present significant challenges to any ICD as rate and morphology discriminators may well classify a bigeminal rhythm as VF due to morphology changes and potentially T wave oversensing of the ectopic beats. This was a particular issue with the early experience in one group of young inherited arrhythmia patients – consisting of 12 patients with primary ventricular arrhythmic disorders: four long QT syndrome (LQTS), three catecholaminergic polymorphic VT (CPVT), three Brugada syndrome, two idiopathic VF and four cases with structural congenital heart disease [27]. Four of these patients experienced a total of ten inappropriate shocks, all due to T-wave over-sensing. There were no significant differences between characteristics of patients with and without inappropriate shocks. In two patients with CPVT, these occurred during frequent polymorphic ventricular ectopy and sinus tachycardia in an Ebstein’s and LQT patient. The median detection rate programmed at the time of the events was 220 bpm (range 190–220 bpm). In two cases a delay in VF therapy occurred due to noise initially detected on the electrogram. Both patients were programmed with a single shock zone from 220 bpm. One episode of VF was quite prolonged before therapy (27 s); analysis revealed that the presence of a conditional sock zone from 200 bpm would have reduced the time to onset of charging from 14 to 9 s. This is because the earlier events in the episode, prior to detection of tachycardia, were sensed with a frequency between 200 and 220 bpm. The presence of a zone from 200 bpm would have therefore caused these earlier events to be classified as tachycardia; however this would increase the risk of T wave oversensing and inappropriate shocks in these cases. This small series highlights the challenges in optimal patient selection for the S-ICD and the need for careful ECG screening at rest and on exercise to minimise the risk of inappropriate shocks in these young often more vulnerable channelopathy patients. However, these issues have not been raised as a major concern in the later experience of larger Dutch series and EFFORTLESS channelopathy populations [11•].

## Limitations of the S-ICD: Bradycardia Pacing and Anti-Tachycardia Pacing (ATP)

The S-ICD system is unable to deliver permanent pacing or ATP to terminate VT. It can deliver 30 s of transthoracic post-shock pacing if the patient is severely bradycardic. Therefore, it cannot be employed in patients with pacing indications. This raises the question as to what proportion of patients who would qualify for an S-ICD would ultimately require pacing or ATP & hence need conversion of their device to a transvenous system. A recent Dutch study of ICD recipients reviewed 463 patients followed up over a median of 3.4 years between 2002-11 [28]. It was demonstrated that 55.5 % (95 % CI 52.0 % to 59.0 %) of single or dual chamber ICD recipients would have been suitable for an initial S-ICD implantation after 5 years, i.e. did not develop a pacing indication or receive ATP from their device. Significant predictors for the unsuitability of an S-ICD were: secondary prevention, severe heart failure and prolonged QRS duration.

The risks and benefits of ATP to terminate VT have come into sharp focus recently from the Reduction in Inappropriate Therapy and Mortality through ICD Programming (MADIT-RIT) study which randomised 1500 patients to three treatment strategy arms: (i) A control group was programmed to treat rates >170 beats per minute with a short delay after initial detection and multiple ATP sequences in the lower rate zone (ii) single zone of therapy at 200 beats per minute with conventional detection delay and ATP while charging or (iii) three-zone therapy arm with prolonged delays; both included use of ATP. Both arms (ii) and (iii) were associated with a significant reduction in appropriate and inappropriate ICD therapy [29•]. In addition to more ICD shocks, there was a significant three-five fold greater use of ATP in the conventional programming control arm versus the comparator arms. Importantly, the patients randomised to the single zone, high rate programming strategy treating only high ventricular rates had a significant reduction in all-cause mortality when compared with those of the conventional arm. The simpler programming strategy reflects the S-ICD programming platform of high-rate zones of therapy and prolongation of the time from detection to shock delivery, thereby minimising unnecessary ICD therapy [17••], however direct comparisons cannot be made as the S-ICD system does not have the capacity to perform ATP pacing while charging. In the S-ICD IDE trial, the time to therapy for appropriate shocks was 14.6 ± 2.9 s which is within the range of prolongation in detection shown to be beneficial in MADIT-RIT. However, debate continues regarding the risk versus benefits of prolonged detection facilitating self-termination of VT/VF episodes and potentially reduced mortality from unnecessary ATP and shocks versus a potentially higher risk of syncope or avoiding shocks through pace termination of VT [16]. Of note however, in the ALTITUDE Registry, there was a recognised increase in mortality for patients receiving ATP accelerating VT (hazard ratio, 3.03; 95 % confidence interval, 2.65–3.46) [30].

A further point is the proportion of patients likely to develop monomorphic VT that is pace-terminable. In the SCD-HeFT ICD study of 811 patients, 182 (22 %) received at least one ICD shock for VT or VF- 50 % (12 % of the 811) had at least one episode of de novo VF which was not preceded by any VT, while two-thirds of the 182 patients (15 % of 811) had one or more episodes of monomorphic VT (>188 bpm) [31•]. Therefore, it could be estimated that ≈15 % of patients with moderate heart failure may experience high rate monomorphic VT during the first few years following an ICD. Over 45.5 months of follow up, only 1/3 of the patients with VT had more than a single episode in SCD-HeFT, representing only 7 % of the 811 ICD patients in the trial, or a 1.8 % per year risk which is less than the reported risk of transvenous lead failure [17••].

## Complications

The main complications of the S-ICD system have related to infection and suboptimal lead position/movement. In IDE & EFFORTLESS all complications were grouped as follows: all device related (type I), labelling-related (type II), and procedure related (type III). In IDE, the 180-day type I-III complication-free rate was 92.1 % with a lower confidence limit of 88.9 %, above the pre-specified performance goal [4••, 5••]. In EFFORTLESS, 15 system related complications in 14 patients (3 %) occurred in the first 30 days post implant, which accounts for a peri-operative complication-free rate of 97 % [6••]. At 360 days post implant, the documented system or implantation-related complication-free rate was 94 %.

Either suspected or confirmed infections were reported in 5.7 % of IDE patients and 4 % of EFFORTLESS, the majority responding to antibiotics for superficial wound involvement. However, 1.3 % of cases required explanation in IDE and 2.2 % in EFFORTLESS. It is thought that the majority of these infections may relate to initial inexperience with surgical technique of implantation and certainly as physician experience grew and rigorous procedural attention to technique was developed, infection requiring explanation declined in IDE. This early experience of higher initial complication rates was pointed out in the Dutch cohort which then demonstrated significant reductions both in inappropriate shocks and device related complications over time [11•]. Many of these were correctable by optimising screening for T wave oversensing on exercise, utilising a suture sleeve to prevent lead migration and reductions in implant time reducing infection rates with increased implanter experience and improvements in technique. Indeed a recent development by the Dutch group avoids the 3rd superior incision to anchor the lead by simply tunnelling the lead through a splittable sheath and anchoring it inferiorly with the suture sleeve [7•]. Lead stability is maintained and the additional incision with extra infection risk and its cosmetic impact avoided.

## Conclusion and Future Directions

Early experiences highlight the S-ICD as a viable alternative to conventional transvenous ICD implantation in patients who do not have a requirement for pacing. Its major advantage is that it negates the risks associated with transvenous lead placement, avoiding acute and chronic lead complications. This has genuine advantages in patients who are young, where longevity of transvenous leads is a real concern. This technology therefore has the potential to change and expand the use of ICDs. Although initial trials show a comparable defibrillation success rate and similar rates of inappropriate therapy to conventional systems, there will be improvements in the device’s surface analysis of heart rhythm; as has already occurred in the lifespan of both the IDE [4••] and EFFORTLESS [6••] studies; where the incidence of oversensing has been reduced by dual zone programming. Further developments in the device’s programming will no doubt refine rhythm discrimination. A better understanding of its role in young patients with inherited arrhythmia disorders and cardiomyopathies such as Brugada, long-QT and HCM, through larger studies are needed along with the programming optimisations that these patients may require.

There is a vital need to continue to monitor the device’s long term performance, safety and efficacy. At present studies have focussed on early experience. However, a randomised controlled trial (The Netherlands-based Prospective, RAndomizEd comparison of subcuTaneOus and tRansvenous ImplANtable cardioverter-defibrillator therapy study (PRAETORIAN) comparing subcutaneous ICD and trans-venous ICD systems in suitably indicated patients is in progress and should allow contemporary comparison between the systems on a number of levels [32]. Next generation S-ICD systems will be reduced in volume to facilitate implantation in thinner patients and paediatric cases. Wireless communication will facilitate remote follow-up. There are also rapid developments occurring in the field with the deployment of leadless electrodes (Nanostim) for pacing which will potentially widen the indication for S-ICD patients to incorporate patients requiring pacing provided oversensing challenges can be overcome. Since paced patients have been successfully implanted with the S-ICD these should be manageable.

Early work is therefore clearly positive in showing that this device compares favourably with conventional ICD systems. As further long term studies and improvements to the device are made, the scope of the S-ICDs future role will become clear.
